# Editorial: Advancing understanding of blast disease: pathogen genomics and host-pathogen interactions

**DOI:** 10.3389/fpls.2025.1754950

**Published:** 2025-12-11

**Authors:** Bochra A. Bahri, Richard A. Wilson, Jauhar Ali

**Affiliations:** 1 Department of Plant Pathology, Institute of Plant Breeding Genetics and Genomics, University of Georgia, Griffin, GA, United States; 2 Department of Plant Pathology, University of Nebraska-Lincoln, Lincoln, NE, United States; 3 Rice Breeding Innovation Department, International Rice Research Institute, Los Banos, Laguna, Philippines

**Keywords:** disease management, ferroptosis, pathogenicity, population genetics, simulation model, Tet-Off system, wheat and rice blast

Blast disease, caused by the fungus *Magnaporthe oryzae* (syn. *Pyricularia oryzae*), poses a significant threat to rice production and to other vital crops—including wheat, barley, millet, and oats—thereby influencing global food security. Although extensive research and breeding programs have aimed to develop resistant cultivars, such resistance typically breaks down within a few years, resulting in severe epidemics. Advances in sequencing technologies have enhanced our insight into the biology and genomics of *M. oryzae*, yet substantial knowledge gaps persist, especially regarding molecular interactions between the pathogen and hosts, and the pathogenicity of *M. oryzae*. The fungus’s genetic diversity and host-specific lineages further complicate management efforts, underscoring the need for comprehensive studies on its infection mechanisms, evolutionary dynamics, and potential for cross-infectivity. By addressing these challenges in this Research Topic, we hope to deepen our understanding of blast disease and support the development of sustainable management strategies.

Rice blast, caused by *Magnaporthe oryzae* pathotype Oryzae (MoO), was first documented in China in the 17^th^ century and is now widespread across more than 100 countries. Although the evolutionary origins and genetic structures of Asian and European *M. oryzae* populations of rice are relatively well understood, African isolates remain largely uncharacterized, limiting the development of effective disease management strategies in the region. By analyzing 180 genome sequences, including 45 from African countries, Onaga et al. showed that migration, founder events, and region-specific adaptations have collectively shaped *M. oryzae* populations in sub-Saharan Africa. *M. oryzae* was probably introduced into Africa primarily from China in the late 19^th^ century, first appearing in West Africa (Mali and Burkina Faso), then spreading to Uganda and Madagascar in the early 20th century, before gradually reaching other parts of the continent through repeated introductions. Gene flow within sub-Saharan Africa links regional *M. oryzae* populations, while effector gene diversity reflects a combination of conserved virulence mechanisms and adaptation to local hosts. Similarly, wheat blast, caused by *Magnaporthe oryzae* pathotype Triticum (MoT), was first reported in Brazil in 1985 and subsequently spread to Bolivia, Paraguay, and parts of Argentina. Its initial occurrence in South Asia was recorded in 2016, where it affected wheat production in Bangladesh. During the 2017–2018 growing season, it appeared in Africa for the first time, disrupting wheat cultivation in Zambia. To safeguard global rice and wheat production from the continually evolving threats posed by *M. oryzae*, it is essential to survey the pathogen’s population dynamics and to develop integrated management strategies that account for both historical and contemporary patterns across continents.

Since these outbreaks, rice and wheat blast have remained a persistent concern, with significant potential for yield losses and economic impact under favorable climatic conditions. Ragulakollu et al. highlighted recent advances in rice blast management, particularly molecular breeding strategies for blast-resistant rice, including marker-assisted selection, marker-assisted backcrossing, transgenic approaches, and genome editing. The review underscored the importance of an integrated approach to enhance resistance durability and ensure the protection of global rice production against *M. oryzae*. For instance, coupling *Mo* life cycle simulation models with crop modeling can strengthen decision support tools to aid agricultural decision-making. Krupnik et al. simulated the effects of wheat blast inoculum build-up on grain yield using a generic disease model and analyzed their occurrence across four varieties and five sowing dates in Bangladesh over the 23 years from 2001 to 2023. Their findings show that delayed sowing is associated with lower yields and greater disease incidence due to elevated temperatures and relative humidity, and that wheat varieties with blast resistance can effectively mitigate yield losses.

Improving our understanding of plant–*M. oryzae* interactions may also provide new tools for integrated disease management. Advances in gene-knockout techniques have simplified the functional validation of genes associated with pathogenicity. Deng et al. revealed that mutations in the *PMK1* and *MAC1* genes are key contributors to the loss of virulence in strain AM16 of rice blast. AM16 transformants overexpressing the *PMK1*^(Guy11)^ and/or *MAC1*^(Guy11)^ alleles from the Guy11 strain exhibited markedly increased conidiation, functional appressorium formation, and restored pathogenicity on rice. Ferroptosis—an iron-dependent regulated cell death mechanism— has also recently been recognized as a crucial mechanism in the pathogenesis of *M. oryzae.* The iron–copper chaperone Ict1 and the copper transporter Ccc2 were identified as key contributors to ferroptosis in the rice blast fungus. Mutants exhibited reduced iron accumulation in conidia and suppressed cell death, defects that were restored mainly by exogenous ferric ions and, to a lesser extent, by copper (Shen et al.). Santoni et al. synthesized current knowledge on ferroptosis mechanisms—particularly iron metabolism and lipid peroxidation—and assessed the potential of antioxidants, including the natural flavonoid tangeretin, to inhibit this pathway as a strategy for managing rice blast disease and enhancing sustainable rice production. Studying gene function in *M. oryzae* can be particularly challenging. Considerable time and effort are invested in the gene knockout process, especially for vital genes, yet it often fails to yield the desired transformants. Recently, the Tet-Off system, based on doxycycline or tetracycline-driven transcriptional repression, provided a powerful approach to study gene function in *M. oryzae* without requiring complete gene deletions, thereby enhancing our understanding of their roles in fungal development and pathogenesis with a simple, reproducible, and rapid method. Fang et al. developed the Tet-Off system to control gene expression in *M. oryzae*. It successfully validated the functions of genes required for hyphal growth, highlighting its utility for studying genes essential for pathogenicity.

In summary, this Research Topic aims to bring together the most recent advances in epidemiology, pathogenicity, genomics, and management of blast disease in rice and wheat. Effective disease management requires holistic approaches such as breeding durable, blast-resistant, and climate-resilient varieties, predicting disease and pathotype dynamics and emergence, improving planting schedules, water and nutrient-use efficiency, and weed control, and judiciously using modern fungicides, plant defense activators, and biological control agents. By implementing these key agronomic management techniques, a practical, sustainable, and long-term blast control could be achieved.

